# Falling-down bladder flap reconstruction for recalcitrant bladder neck contracture: One complex case and stepwise technique

**DOI:** 10.1016/j.eucr.2026.103435

**Published:** 2026-04-09

**Authors:** Tarek Taha, A. Ami Sidi, Alexander Tsivian

**Affiliations:** a Department of Urologic Surgery, Wolfson Medical Center, Holon, Israel; b Gray Faculty of Medical and Health Sciences, Tel Aviv University, Israel; c Department of Urologic Surgery, Ziv Medical Center, Safed, Israel; d The Faculty of Medicine in the Galilee, Bar-Ilan University, Israel

## Abstract

This study describes a transvesical “falling-down” anterior bladder wall flap reconstruction for recalcitrant bladder neck contracture (BNC). One complex case, a rotated, well-vascularized bladder flap was sutured to the distal urethra/prostate bed after wide bladder exposure. A 49-year-old man underwent combined procedures with diverticulectomy and ureteral reimplantation, achieving normal uroflow (Qmax 17 mL/s) and no residual urine for three years. The falling-down bladder flap offers durable tissue and wide exposure to facilitate concomitant repairs and may complement traditional BNC techniques.

## 1 Introduction

Bladder neck contracture (BNC)—also referred to as bladder neck obstruction (BNO) in some reports—is a recognized complication following transurethral instrumentation such as transurethral resection or vaporization of the prostate (TURP/TUVP).^1^ Reported rates vary widely by technique and cohort^2^, ^3^, ^4^. Clinically, patients typically present with lower urinary tract symptoms consistent with bladder outlet obstruction, including weak stream, hesitancy, incomplete emptying, or recurrent catheterization. Diagnosis relies on careful clinical assessment supported by uroflowmetry, post-void residual measurement, urodynamics when indicated, and cystoscopy for direct visualization of the narrowed bladder neck.^5^ While endoscopic incision/dilation is often first-line, refractory cases may require open or robotic reconstructive surgery. Here we present two complex cases managed since 2008 using a transvesical falling-down bladder flap technique and detail the operative steps, nuances, and outcomes.

## 2 Surgical technique: falling-down rotated bladder flap

Patient positioning and access: After standard preparation, a midline longitudinal cystotomy is performed on the anterior bladder wall and extended until adequate bi-valving exposure is achieved. The bladder is inspected thoroughly to identify concomitant pathology (e.g., diverticula) and the true urethral lumen.

Flap design: A rectangular/triangular anterior bladder wall flap is mobilized with an approximately 5 cm base (between points B and C) and 3 cm width at the apex (point A), with a length of 5–6 cm. One lateral margin of the flap coincides with the primary cystotomy and is continued through the bladder neck toward the urethra.

**Fig. 1:**
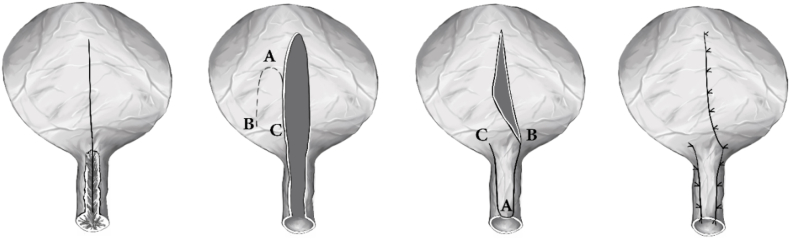
**Falling-down rotated bladder flap technique (schematic).** A. A midline longitudinal cystotomy is performed on the anterior bladder wall and extended to the bulbar urethra. B. A rectangular/triangular anterior bladder wall flap is mobilized and lumen identified. C. The flap is elevated and rotated downward. The flap is prepared to fall into place and cover or reconstruct a defect. D. Inset and final alignment.

Lumen identification: The urethra is exposed both retrogradely and antegradely (from the bladder) as needed, especially when false passages are present. Flap rotation and inset: The flap is rotated inferiorly ("falling-down") and the apex sutured to the lowest point of the urethra or to the prostate bed in men, using interrupted absorbable sutures. The remaining flap is laid anteriorly along the urethral segment to augment/replace the scarred bladder neck and provide a well-vascularized lining. Fig. 1.

Drainage and imaging: A urethral catheter and, when indicated, a suprapubic catheter are left in situ. Voiding cystourethrography (VCUG) at approximately two weeks confirms integrity and patency prior to catheter removal.

## 3 Case presentation

**Fig. 2:**
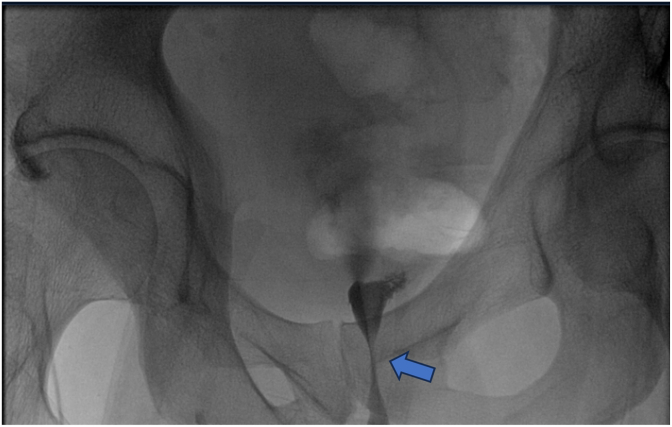
Retrograde urethrography (RUG): passage of contrast from the prostate bed to the bladder through a pinpoint aperture (arrow).

**Fig. 3:**
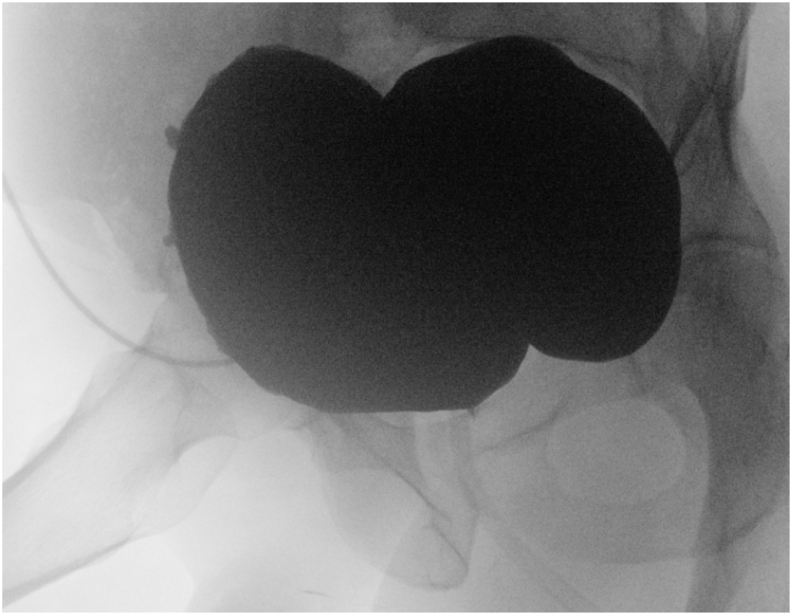
Voiding cystourethrography (VCUG) prior to surgery demonstrating a large bladder diverticulum and no passage to the prostate bed.

**Fig. 4:**
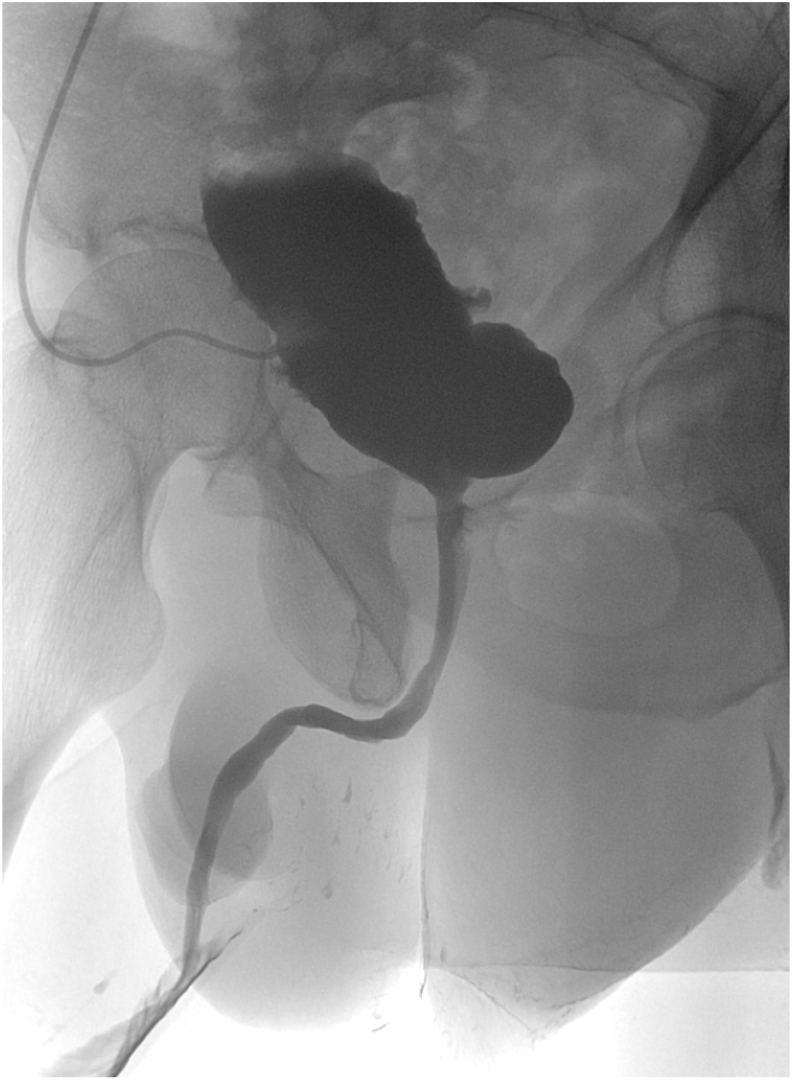
Postoperative VCUG confirming patency and absence of leakage.

A 49-year-old man developed urinary retention several months after TURP. Initial retrograde catheterization was difficult; a suprapubic catheter (SPC) was placed. Cystoscopy showed BNC and he underwent three endoscopic bladder neck incisions/resections without durable relief (Fig. 2). Subsequent evaluation revealed a concomitant recurrent bulbar urethral stricture and a large bladder diverticulum (Fig. 3). He underwent internal urethrotomy, diverticulectomy with ureteral reimplantation (left ureter inserted into the diverticulum), and falling-down bladder flap reconstruction of the bladder neck. The midline bladder incision was continued through the fibrotic, narrowed bladder neck to the prostate bed, and the flap apex was sutured at that level. There were no intraoperative complications. VCUG at two weeks (Fig. 4) showed no leakage; the ureteral stent was removed at six weeks. Uroflowmetry at one and three months demonstrated Q max 17 mL/s with no post-void residual. Over three years of follow-up he reported no urinary symptoms or complications.

## 4 Discussion

Recalcitrant BNC after transurethral instrumentation poses a reconstructive challenge, particularly when concurrent urethral and bladder pathology is present^6^, ^7^, ^8^. The goals of reconstruction is to establish a patent outlet, preserve continence when achievable, minimize recurrence, and protect upper tract function. Traditional options such as Y–V plasty can be effective for isolated BNC(^9^) but may limit exposure when extensive scarring, false passages, or concomitant lesions require correction. Buccal mucosa grafts provide versatile tissue for urethral reconstruction,^10^ yet their behavior under bladder outlet pressures and risk of edema or contracture at the bladder neck may be suboptimal in selected scenarios. In contrast, local bladder wall flaps offer robust, well-vascularized tissue with minimal donor-site morbidity and can be integrated seamlessly into transvesical approaches. In our complex case, broad bladder exposure facilitated precise identification of the true urethral lumen and correction of associated pathology, while the falling-down flap provided durable lining and caliber. The favorable functional outcomes—catheter independence in our patient, normal uroflow, and absence of recurrent obstruction during follow-up—support this technique as a valuable option for carefully selected patients. Limitations include the small sample size and retrospective nature; larger series and longer follow-up are warranted to refine indications and compare outcomes with alternative reconstructions.

## 5 Conclusion

A falling-down rotated bladder flap is a practical reconstructive solution for recurrent, recalcitrant bladder neck contracture. By combining wide transvesical exposure with a well-vascularized local flap, this technique facilitates concomitant repairs and may improve durability in complex anatomy. Careful patient selection and structured follow-up are essential.

## CRediT authorship contribution statement

**Tarek Taha:** Writing – original draft, Data curation, Conceptualization. **A. Ami Sidi:** Writing – review & editing, Investigation. **Alexander Tsivian:** Writing – review & editing, Methodology, Investigation, Conceptualization.

## Funding sources

This research did not receive any specific grant from funding agencies in the public, commercial, or not-for-profit sectors.

## Declaration of competing interests

"I have nothing to declare"

## References

[1] Ramirez D., Zhao L.C., Bagrodia A., Scott J.F., Hudak S.J., Morey A.F. (2013 Dec 1). Deep lateral transurethral incisions for recurrent bladder neck contracture: promising 5-year experience using a standardized approach. Urology.

[2] Primiceri G., Castellan P., Marchioni M., Schips L., Cindolo L. (2017 Oct). Bladder neck contracture after endoscopic surgery for benign prostatic obstruction: incidence, treatment, and outcomes. Curr Urol Rep.

[3] Blaivas J.G., Flisser A.J., Tash J.A. (2004 March). Treatment of primary bladder neck obstruction in women with transurethral resection of the bladder neck. J Urol.

[4] Nitti V.W. (2005). Primary bladder neck obstruction in men and women. Rev Urol.

[5] Huckabay C., Nitti V.W. (2005 Jul). Diagnosis and treatment of primary bladder neck obstruction in men. Curr Prostate Rep.

[6] Castellani D., Stramucci S., Enganti B. (2024 May 7). One-year outcomes of transurethral treatment of bladder neck stenosis following transurethral resection of the prostate. results from a large, multicenter series. Int J Gen Med.

[7] Sussman R.D., Drain A., Brucker B.M. (2019). Primary bladder neck obstruction. Rev Urol.

[8] Cash H., Wendler J.J., Minore A., Goumas I.K., Cindolo L. (2024 Mar). Primary bladder neck obstruction in men—new perspectives in physiopathology. Prostate Cancer Prostatic Dis.

[9] Sayedahmed K., El Shazly M., Olianas R., Kaftan B., Omar M. (2019). The outcome of YV plasty as a final option in patients with recurrent bladder neck sclerosis following failed endoscopic treatment. Central European J Urology.

[10] Markiewicz M.R., Lukose M.A., Margarone J.E., Barbagli G., Miller K.S., Chuang S.-K. (2007 Aug). The oral mucosa graft: a systematic review. J Urol.

